# Q-DEPICT: Qatar Determining Emergency Physician Incidence of COVID-Positive Testing

**DOI:** 10.5339/qmj.2021.44

**Published:** 2021-09-29

**Authors:** Shada A. Kodumayil, Ashid Kodumayil, Sarah A. Thomas, Sameer A. Pathan, Zain A. Bhutta, Isma Qureshi, Aftab Azad, Tim R. Harris, Stephen H. Thomas

**Affiliations:** ^1^A-level candidate, Doha College, Doha, Qatar; ^2^Department of Emergency Medicine, Hamad General Hospital, Doha 3050, Qatar E-mail: ZBhutta@hamad.qa; ^3^BSc Candidate in Medical Biosciences, Faculty of Medicine, Imperial College London, UK; ^4^School of Public Health and Preventive Medicine, Monash University, Melbourne, Australia; ^5^Blizard Institute, Barts and The London School of Medicine, Queen Mary Univ. of London, UK; ^6^Doctoral School of Health Sciences, University of Helsinki, Helsinki, Finland

**Keywords:** emergency medicine, COVID-19, Testing, Prediction model, Qatar

## Abstract

Despite protective measures such as personal protective equipment (PPE) and a COVID airway management program (CAMP), some emergency physicians will inevitably test positive for COVID. We aim to develop a model predicting weekly numbers of emergency physician COVID converters to aid operations planning.

The data were obtained from the electronic medical record (EMR) used throughout the national healthcare system. Hamad Medical Corporation's internal emergency medicine workforce data were used as a source of information on emergency physician COVID conversion and numbers of emergency physicians completing CAMP training. The study period included the spring and summer months of 2020 and started on March 7 and ran for 21 whole weeks through July 31. Data were extracted from the system's EMR database into a spreadsheet (Excel, Microsoft, Redmond, USA). The statistical software used for all analyses and plots was Stata (version 16.1 MP, StataCorp, College Station, USA). All data definitions were made *a priori*.

A total of 35 of 250 emergency physicians (14.0%, 95% CI 9.9%–19.9%) converted to a positive real-time reverse transcriptase-polymerase chain reaction (PCR) during the study's 21-week period. Of these. only two were hospitalized for having respiratory-only disease, and none required respiratory support. Both were discharged within a week of admission. The weekly number of newly COVID-positive emergency physicians was zero and was seen in eight of 21 (38.1%) weeks. The peak weekly counts of six emergency physicians with new COVID-positive were seen in week 14. The mean weekly number of newly COVID-positive emergency physicians was 1.7 ± 1.9, and the median was 1 (IQR, 0 to 3).

This study demonstrates that in the State of Qatar's Emergency Department (ED) system, knowing only four parameters allows the reliable prediction of the number of emergency physicians likely to convert COVID PCR tests within the next week. The results also suggest that attention to the details of minimizing endotracheal intubation (ETI) risk can eliminate the expected finding of the association between ETI numbers and emergency physician COVID numbers.

## Introduction

The Severe Acute Respiratory Syndrome Coronavirus-2 (SARS-CoV-2), through its associated disease COVID-19 (hereafter, COVID), has had a significant impact on Emergency Department clinical practice worldwide, including Qatar's national healthcare system Hamad Medical Corporation (HMC). Unlike most operations stressors in emergency medicine (EM), COVID poses risks to ED physicians (EPs). Risk remains even with measures such as patient isolation and the use of personal protective equipment (PPE).^1,2^

Mitigation plans for occupational risks of EPs contracting COVID include such measures as instituting locked-down COVID isolation areas and requiring universal PPE (*e.g.,* gowns, N-95 masks).^3^ In an attempt to mitigate specific risks associated with endotracheal intubation (ETI), Qatar's national-level EM department implemented a COVID airway management program (CAMP) across the country's EDs. The COVID Airway Management Training was developed to improve the skills and safety of a volunteer core of EPs who delivered rapid sequence intubation (RSI) in the ED during the pandemic. It consists of lectures on COVID-adapted RSI, safe procedural sedation, mechanical ventilation, human factors and team working, MCQ tests (RSI, mechanical ventilation), online course on mechanical ventilation, motor skills workshop on mechanics of intubating with Mac 4, video (Glydescope), video (CMAC), bougie use, stylet use, use of and training in the use of dedicated ad hoc documentation (to standards of American NEAR database) and a simulation (4 hours, half-day) to develop communication skills.

Despite protective measures such as PPE and CAMP, some EPs will test positive for COVID. In Qatar's EDs, positive COVID results on polymerase chain reaction (PCR) testing require time out of work. Personnel pressures associated with COVID are substantial, and ED operations planners stand to benefit from predicting weekly numbers of EPs who will become newly PCR-positive.

The primary aim of this study, the **Q**atar **D**etermining **EP I**ncidence of **COVID**-positive **T**esting (Q-DEPICT) develops a model predicting weekly numbers of EP COVID converters to aid operations planning. A secondary aim was to ascertain whether the factors influencing EP COVID numbers included weekly counts of ETI or noninvasive ventilation (NIV) since these procedures were thought to carry a particular risk of occupational COVID transmission.

## Methods

### Design

This study was primarily sourced using routinely collected ED data in the EMR system (FirstNet, Cerner, Kansas City, USA) used throughout the national healthcare system. Internal HMC EM workforce data were used to collect EP COVID conversion and numbers of EPs completing CAMP training.

Data were obtained for the spring and summer of 2020. During this time frame, COVID emerged, peaked, and declined in Qatar's EDs. The study period commenced on March 7 (the day after HMC's first ED patient COVID diagnosis) and ran for 21 whole weeks through July 31. The demonstration that this 21-week period included Qatar EDs’ rise and fall of COVID is found in Supplementary file: Appendix 1.

Data were extracted from the system's electronic medical record database into a spreadsheet (Excel, Microsoft, Redmond, USA). The statistical software used for all analyses and plots was Stata (version 16.1 MP, StataCorp, College Station, USA). All data definitions were made *a priori*.

### Setting

The study was set in Qatar's national healthcare system, HMC. HMC operated three EDs during the country's COVID epidemic, from March through July. Hamad General Hospital (HGH) ED is viewed as HMC's Doha tertiary care center. HGH ED historically sees approximately 400,000 cases annually. Al Wakra Hospital (AWH) ED is located approximately 10 miles south of Doha and has an annual volume of approximately 210,000 patients. Al Khor Hospital (AKH) ED, located approximately 30 miles north of Doha, has an annual volume of approximately 160,000 patients. The weekly census of all study EDs dropped during the COVID period.

The HMC study EDs are all administered by a centralized EM Department. Across the system, COVID infection control precautions were instituted in March and maintained throughout the study period. Lower-acuity (walk-in) cases with COVID risk factors (*e.g.*, fever, respiratory complaints, travel history, known exposures) were directed from outdoor triage areas to nearby COVID evaluation tents. Higher-acuity cases, such as those arriving by ambulance (EMS), underwent similar “front-door” screening and were directed to dedicated (access-controlled) COVID areas if they had risk factors. All cases requiring major resuscitation were treated as if they were COVID-positive.

The EDs’ infection control arrangements were constant during the study period. The only ED-related occupational risk parameter that changed during the March–July time frame was CAMP. Due to the size of the EP group, CAMP was rolled out continuously. The first EMPs were trained at the end of March, and training continued throughout the summer. By June, program completion became a requirement for all EPs providing ETI. The evolution of the CAMP training numbers over the study period is shown in Supplementary file: Appendix 1.

CAMP targeted the approximately 250 EPs providing care in the country's EDs. AKH and AWH EDs were staffed by board-certified Emergency Medicine Specialists and Consultants, who also rotated to provide care at HGH. HGH ED serves as the HMC EM teaching locus, where consultant and specialist care is augmented by 40 EM residents (in a five-year training program) and 14 EM fellows (in a two-year training program). Residents and fellows work only at HGH, but because of COVID-related staffing pressures, consultants and specialists continued to rotate among the HMC EDs.

Daily coverage patterns called for approximately 80, 35, and 20 total EP shifts per day at HGH, AWH, and AKH, respectively. The targeted weekly EP coverage level of 945 shifts (*i.e.*, daily coverage of 135 for seven days) fluctuated. Supplementary file: Appendix 1 provides information on the fluctuation in weekly EP coverage levels.

COVID testing for HMC EDs is centralized and uses PCR technology. All EDs in the HMC system had COVID testing performed at the country's virology laboratory on HMC's main campus in Doha. The specifics of sample handling and PCR methods used by HMC clinical laboratories have been described in detail elsewhere.^4^

As described in Supplementary file: Appendix 2, the admitted case *n* was included in the model. However, *p* exceeding 0.05 is supported by *a priori*-defined criteria, including Akaike's information criteria, Bayesian information criteria, and Pearson deviance. Supplementary file: Appendix 2 includes additional information on variables that were not found significant in modeling. Most important among these was the weekly number of ETIs. ETI numbers were not associated with EP COVID counts when added to Table 1variables (*p* = 0.239) or when ETI count replaced the NIV count (*p* = 0.696).

**Figure 1.**
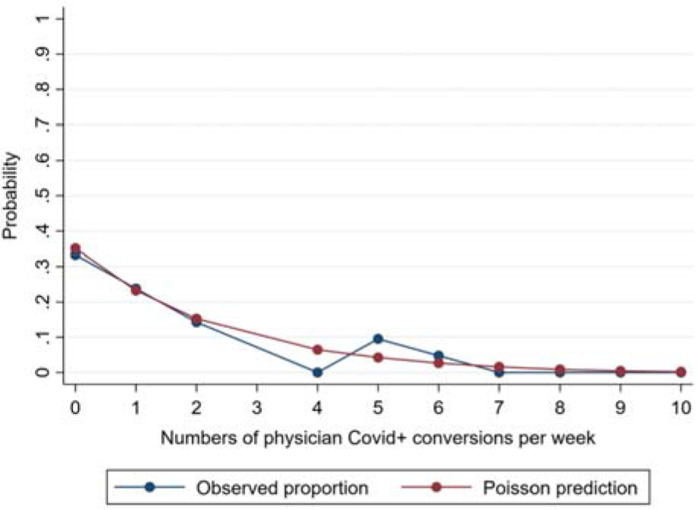
Poisson regression-predicted vs. actual weekly counts of EP COVID conversion

The performance of the model containing Table 2 variables was acceptable using standard measures of classification, calibration, and deviance.^5^ The classification across different predicted probabilities is suggested by the model-predicted *vs.* actual proportions of weekly COVID counts (Figure 1). Calibration was assessed as acceptable by a postestimation goodness-of-fit test (*p* = 0.458). The specification was acceptable, as indicated by finding a Pearson deviance of 1.00 (nonrounded Pearson deviance = 0.995).

### Analysis

Q-DEPICT's unit of analysis was the study week (*n* = 21). Unless otherwise noted, descriptive and analytical statistics focused on weekly numbers rather than daily or per-shift numbers.

For continuous data, descriptive assessment commenced with the assessment of normality using formal statistical tests (Shapiro-Wilk test) and visualization with normal quantile plots. Normal data's central tendency and dispersion are described using the mean ± standard deviation (SD); nonnormal data are described using the median and interquartile range (IQR). Univariate comparisons for continuous data were performed with Kruskal-Wallis nonparametric tests.

Categorical data are described using proportions. Binomial exact 95% confidence intervals (CIs) were calculated for key proportions (*e.g.,* % of workforce EPs with positive COVID PCR). Univariate categorical analyses were executed using Pearson χ^2^ tests.

Since there were two predictors, a weekly ED census and a weekly number of ED COVID patients, model-building commenced with a bivariable approach and proceeded using purposeful selection.^9^ Individual covariates’ contributions and appropriateness for inclusion in the final model were assessed using a likelihood ratio test (for nested models), Akaike's and Bayesian information criteria (to compare both nested and nonnested models), and the Pearson dispersion statistic (to assess whether the addition of a covariate improved or worsened model specification).^7^

Since the ultimately selected model approach was the multivariable Poisson regression, results are reported as the incidence rate ratio (IRR) with 95% CIs. The incidence rate ratio reports the proportional change in the incidence of the EP COVID count associated with a one-unit change in the predictor variable of interest; the IRR's null value is 1.0.

## Results

### ED census and patient characteristics

During the 21-week study period, a total of 225,448 patients were seen in HMC EDs. The weekly census over the study period varied between 7528 and 16,138, with a normal distribution (Shapiro-Wilk *p* = 0.320) and mean 10,736 ± 1983.

### Weekly census of study EDs

**Figure 2.**
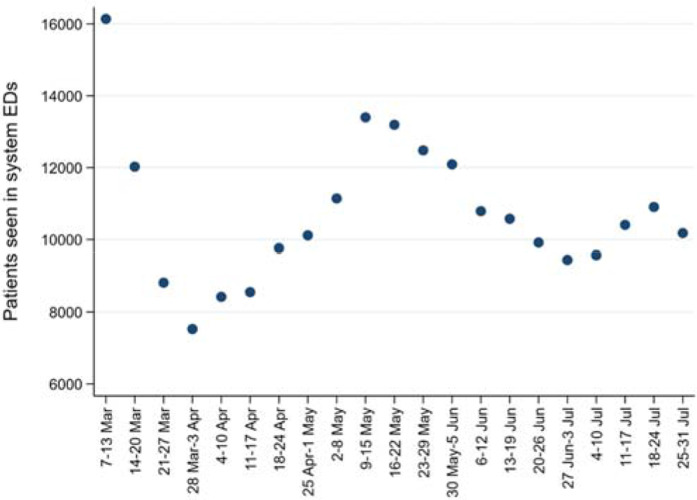
Weekly census in study EDs

The weekly census of the study EDs was lower than the EDs’ usual census in preCOVID years. Weekly census numbers for the study period are shown (Figure 2). The first week's census (over 16,000) is approximately equal to the usual weekly system ED census from previous (preCOVID) years.

During COVID, as at other times, Qatar's study ED sees a predominance of relatively young males; this reflects the national demographics in a country with a large expatriate male workforce. General characteristics of Q-DEPICT's 225,448 patients are shown in Table 1. Supplementary file: Appendix 2 provides a table detailing the weekly descriptive results for each of these variables.

### Weekly census of COVID patients in ED

**Figure 3.**
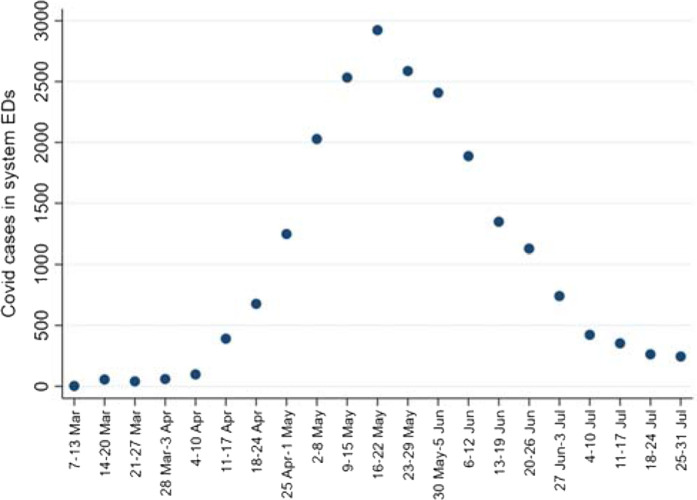
New COVID case diagnoses in study ED patients

It is necessary to include periods during the rise and fall of the COVID epidemic to generate the most useful modeling of EP COVID positivity rates. Figure 3 demonstrates the timing of the COVID epidemic's rise and fall as tracked in ED patients in Qatar. The plot commences with the initial ED diagnosis of COVID in early March and continues through the ED COVID peak in the third week of May. The plot ends in the last week (*i.e.*, the whole last week of July); by this time, the epidemic had waned.

Given the large *n* of study cases (225,448), univariate analyses of the Table 1 patient characteristics were characterized by high precision and statistical significance; all Table 1 variables had *p* < 0.001 for variation with study week. While these findings’ magnitudes were not always of obvious practical significance, each of these patient characteristics was explored for inclusion in multivariable modeling.

### EP weekly COVID conversions

**Figure 4.**
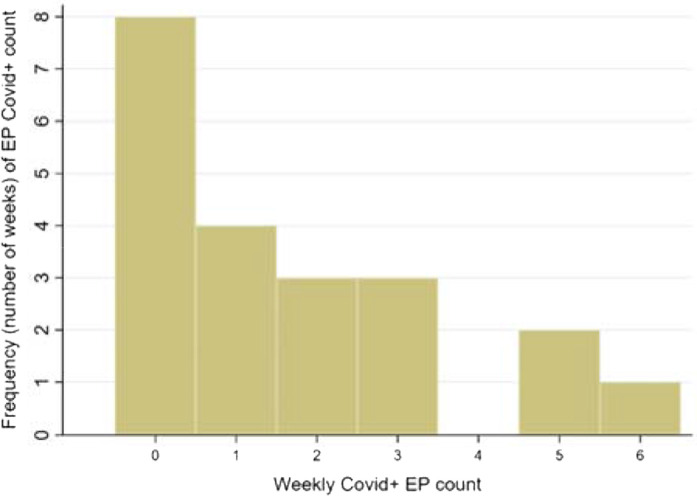
Frequency histogram of EP weekly COVID-conversion counts

A total of 35 of 250 EPs (14.0%, 95% CI 9.9%–19.9%) converted to positive PCR during the study period (Figure 4). Only two were hospitalized for having respiratory-only disease; neither required respiratory support (*e.g.*, intubation), and both were discharged within a week of admission. The weekly modal number of newly COVID-positive EPs was zero, seen in eight of 21 (38.1%) weeks.

The peak weekly count of six EPs with new COVID positivity was seen in week 14 (*i.e.*, 14 weeks after the first COVID-positive ED patient diagnosis). A longitudinal plot of weekly EP COVID conversion counts is shown in Supplementary file: Appendix 2.

The weekly number of COVID-positive EPs was not normally distributed, as demonstrated by Shapiro-Wilk *p* = 0.011 and suggested by the histogram (Figure 4) and quantile-normal plot (Supplementary file: Appendix 2). The mean weekly number of newly COVID-positive EPs was 1.7 ± 1.9; the median was 1 (IQR, 0 to 3).

### Endotracheal intubation and noninvasive ventilation

Over the study period, approximately equal numbers of patients underwent ED ETI (with or without preceding *NIV*) and those who received *NIV* only (*i.e.*, *NIV* was administered, and the patient did not undergo ED ETI). A total of 323 patients underwent ED ETI, and 327 cases received *NIV*.

The weekly number of ETIs was normally distributed (*p* = 0.802) across the 21 study weeks, with a mean of 15.4 ± 7.1. In the 327 NIV cases, the weekly numbers of *noninvasive ventilation* were normally distributed (*p* = 0.751) across the 21 study weeks, with a mean of 15.6 ± 7.1.

**Figure 5.**
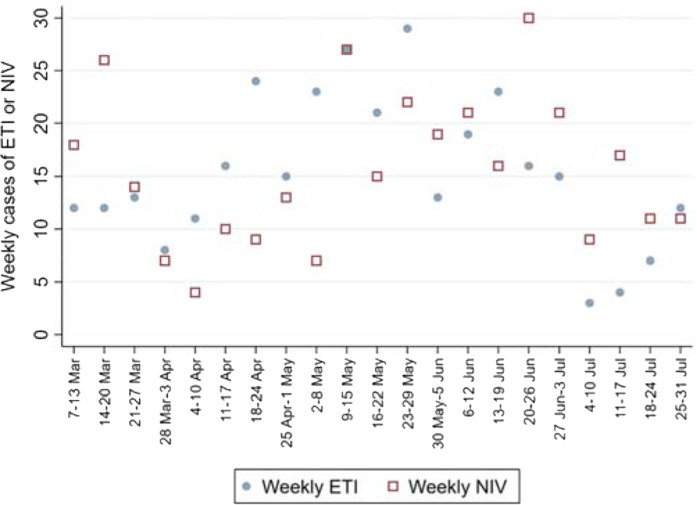
Weekly numbers of patients undergoing ETI or NIV

The weekly case numbers of ETIs and NIV cases are shown in Figure 5. The purpose of the Q-DEPICT was not to assess trends in ETI vs. NIV, but the figure accurately reflects the general practice of EPs in the study system. In the first few weeks of the epidemic, there was a continuation of the EDs’ preCOVID routine of more frequent NIV than ETI. Due to the perceived higher occupational risk associated with NIV than (early, controlled) ETI, HMC ED practice began to favor invasive airway management approximately a month into the epidemic. By the study period's final six weeks the there was a trend toward favoring NIV.

### Modeling weekly EP COVID conversion counts

The variables that were found significantly associated with weekly EP COVID counts, in a model including an offset term for the number of weekly EP clinical shifts (exposures), were the previous week's counts of 1) overall census, 2) COVID-positive patients, 3) admitted patients, and 4) patients receiving NIV. Table 2 shows the *p* value and estimated effect magnitude (as IRR) for each of these variables with 95% CI. Some variables are scaled (*e.g.*, hundreds of cases) to facilitate IRR interpretation.

**Figure 6.**
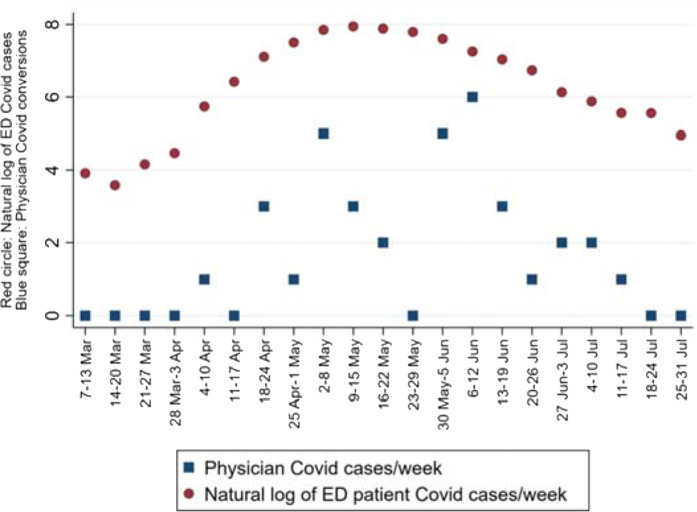
Plot of patient and physician COVID numbers

A plot was constructed to show the chronologies of ED patient COVID counts and EP COVID counts to illustrate the chronologies of both of these variables. Since the EP COVID cases were far fewer than patient COVID numbers, the latter data were log-transformed. This allowed a single plot (with one *y-*axis scale) to depict the patient COVID counts (as a natural logarithm) and EP COVID conversions. The plot (Figure 6) demonstrates that the EPs’ COVID conversion rise and fall generally tracked (with a lag of a week or two) the ED patients’ COVID positivity.

Interpretations of Table 2 findings are as follows: For every additional 1000 patients in the weekly systemwide ED census, there was a 75% decrease in the following week's incidence of EP COVID conversion. In addition, every week's additional 100 COVID-positive ED patients, there was a 20% increase in the following week's incidence of EP COVID conversion. Also, the following week's EP COVID conversion incidence increased by 7% for each NIV case during a given week. Moreover, for each 100 weekly admitted cases (both COVID and other diagnoses) across the system's EDs, the following week's EP COVID conversion incidence increased by 30%.

## Discussion

As 2020 ended, COVID had few proven treatments (except dexamethasone for severe disease).^10^ Also, no vaccine was in widespread use. Therefore, the disease continued to present challenges to EDs in Qatar and around the world. One such challenge was reducing, if not eliminating, the occupational risk to EPs. In addition to the obvious implications for EP wellness, there are essential operations planning reasons for predicting EP resource impacts from COVID conversion.

Q-DEPICT focuses on the capability of modeling EP COVID conversions to inform operations planning regarding EP resources. The Q-DEPICT main result is its production of a well-performing model that requires only four “inputs” for a given week – counts of the census, COVID census, admits, and NIV cases – and predicts the following week's rise or fall in EP COVID incidence. This model has now been implemented for use in Qatar's EDs. The model's development methods described here may prove useful for other systems wishing to predict COVID infection's impact on EP resources accurately.

The study findings, as presented in Table 2, are explainable on operations and clinical grounds. The increased risk of EP COVID conversion as a function of increased ED patient COVID prevalence is unsurprising. The related finding of a “protective effect” of the overall ED census most likely indicates that as COVID peaked, nonCOVID ED visits (and the overall census) dropped. The EP COVID count's prediction by admissions is also clinically sensible since admission rates generally track overall ED acuity; the higher the acuity, the more likely there are “sick.” COVID patients undergoing clinical interactions or procedures risk COVID transmission. Among these risky procedures are NIV and ETI, the findings of which are noteworthy, if preliminary.

Both NIV and ETI are known to pose the risk of COVID transmission.^11,12^ Q-DEPICT's identification of NIV as a predictor of EP COVID counts is sensible. NIV entails patient cooperation requirements and other mechanical factors that seem likely to result in aerosolized secretion dispersion. Previous studies have reported an infection rate as high as 90% in healthcare workers during the use of NIV.^11^ Moreover, since patients cannot be “trained,” NIV-associated COVID transmission risk mitigation must be limited to providing protective measures.^13^

COVID transmission risks are also attendant to ETI, but available evidence suggests that careful planning and execution of controlled ETI can decrease virus transmission risk.^12^ The Q-DEPICT results suggest that attention to details of minimizing ETI risk can eliminate the expected finding of the association between ETI numbers and EP COVID numbers. While nondefinitive, this finding supports the resource investment (time, equipment, others) attendant to the preparation and delivery of Qatar's COVID airway management program. The current retrospective analysis does not allow definitive conclusions about the COVID airway management program and ETI-associated EP COVID risk reduction. However, the preliminary indications of Q-DEPICT favor the ongoing employment of CAMP-type programs to mitigate EP ETI risk.

Q-DEPICT's goal was to generate a model predicting EP COVID conversion, not to assess differential risks of NIV *vs.* ETI specifically. Limitations preclude overextension of the study's suggestion that NIV may pose more risk to EPs than ETI. First, Q-DEPICT assessed the total numbers of cases with NIV and the total numbers of cases with ETI. Since some of the ETI cases received NIV, there was not a discrete NIV *vs.* ETI assessment. Second, assessing relative risks of NIV and ETI would ideally include “exposure” variables, such as NIV time or peri-ETI difficulties. Q-DEPICT's lack of accounting for these factors means that the suggestion of NIV is riskier (for EP COVID conversion) than ETI is not conclusive. A study focused on the different airway and ventilatory support is required before definitive statements can be made regarding EP safety of NIV compared with ETI.

This study did not attempt to ascertain for each COVID-converting EP which clinical (or even nonclinical) encounter was responsible for COVID transmission. EP COVID contraction from nonclinical sources remains a possibility. Such nonclinical mechanisms would not likely be associated with specific clinical predictors. Thus, it would most likely result in diluting observed predictors’ statistical significance (rather than creating false-positive EP COVID predictors). However, the internal validity threat due to lack of precision in identifying each EP's pathway of COVID contraction must be acknowledged.

Q-DEPICT results prompt questions and directions for future analysis. The need to assess for NIV *vs.* ETI safety has been noted. Knowledge of provider risk should not drive, but could reasonably inform, airway and ventilation decision-making in the COVID era.^14,15^ A follow-up study should also assess the prediction of COVID counts (and the reduction of COVID risk) for nurses, respiratory therapists, and others for whom there is an occupational risk of COVID conversion.

## Conclusion

In conclusion, this study demonstrates that knowing only four parameters allows the reliable prediction of the number of EPs likely to convert COVID PCR tests within the next week. These findings are applied here for COVID resource-allocation planning in Qatar. Also, the methods used to generate the prediction model are potentially useful in other settings. As a preliminary conclusion, the current data suggest that the deployment of a focused airway training program can reduce or eliminate the usual association between ETI and occupational COVID risk. However, NIV may be more refractory to risk mitigation in the State of Qatar's ED system.

## Appendix 1: Methodology

This appendix provides further details regarding the study setting. Information includes the weekly census, weekly COVID-patient census (as defined by new COVID PCR positivity), EP numbers completing the system's CAMP training, and the total number of weekly EP shifts worked across HMC EDs.

### COVID airway management program (CAMP) training in the study EDs

**Figure 7.**
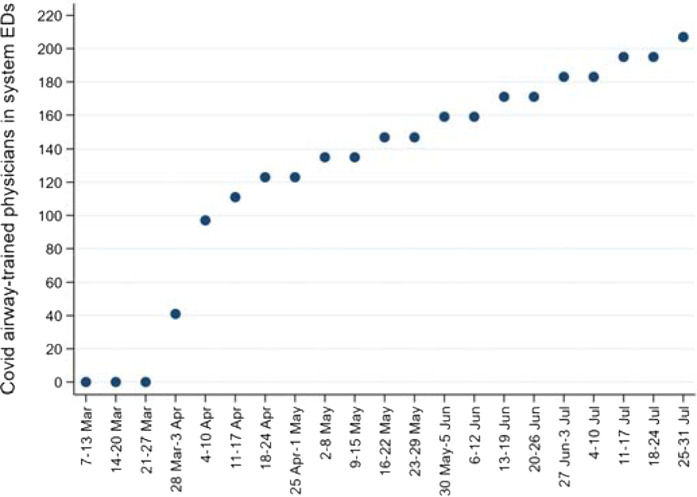
COVID airway management program (CAMP) training in system EDs

The increase in CAMP-trained EPs over the study period is shown in Figure 7. The number of EPs in the country's national healthcare system during the study period fluctuated around 250; by July's end, approximately four of every five EPs had completed CAMP.

### Weekly EP shifts

The daily coverage targets for HGH (80), AWH (35), and AKH (20) sum to 135. Thus, the coverage target for EP shifts across the system was 945 shifts per week. However, during the COVID epidemic, there were EP personnel shortages arising from multiple sources (*e.g.*, required quarantine based on exposure).

The shift target number of 945 was the observed (actual) median of weekly EP shifts during the study period. The mean ± SD was 943 ± 28. Weekly coverage data were distributed normally by the Shapiro-Wilk test (*p* = 0.081) and by quantile-normal plots (not shown).

**Figure 8.**
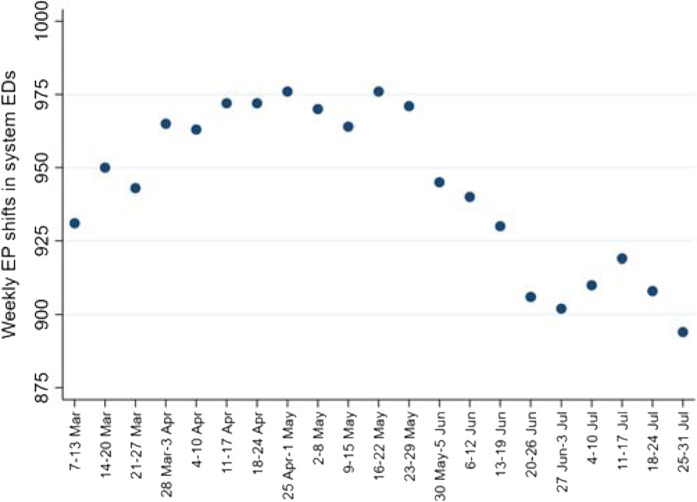
Weekly EP shifts worked across the system

The range of weekly EP shifts varied from a low of 894 to a high of 976 (Figure 8). Based on the difference that ranged to nearly a dozen (11.7) EP shifts per day, a decision was made to incorporate the number of weekly EP shifts into multivariable modeling. The decision was based on the fact that one week's “denominator” of EP shifts risking COVID exposure for occupational transmission could be substantially different from another week's denominator. This variable was thus incorporated into Poisson regression modeling as the offset variable (sometimes known as the exposure variable).^7^

## Appendix 2: Extended Results

### Characteristics of *n*=225,448 study subjects over 21 weeks

The table below depicts each week's findings regarding mean ( ± SD), median (IQR), or proportion of that week's ED patients with demographic characteristics of interest.

**Figure 9.**
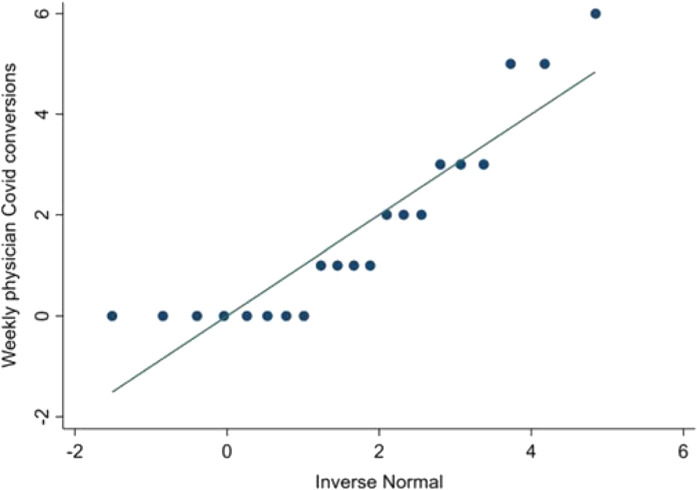
Quantile-normal plot of weekly EP COVID numbers

Weekly EP COVID numbers were not distributed normally. This is demonstrated by the quantile-normal plot in Figure 9.

### Developing the generalized linear modeling for EP COVID conversion

Since the weekly EMP COVID conversion rate's unconditional mean (1.7) was substantially exceeded by its variance (3.5), an unadjusted Poisson model was expected to have a poor fit. This was confirmed by assessing a simple (intercept-only) model with postestimation goodness-of-fit *p* of .003. This portion of the appendix describes the modeling approach using Stata's *glm* and *countfit* procedures. The overall goal was to develop a Poisson count model using predictors of known or theorized importance and then assess whether the Poisson regression model was sufficient or should be replaced with an NB2 model (to handle residual heterogeneity) zero-inflated versions of either Poisson or NB2 regression.

### Terms for the model

Two variables were selected *a priori* for inclusion in the model: weekly census and weekly COVID-positive census (*i.e.*, number of weekly cases of new ED PCR diagnosis of COVID). For ease of interpreting IRRs, the weekly census was analyzed as “thousands of patients/week,” and the weekly COVID-positive caseload was analyzed as “hundreds of COVID-positive patients/week.”

In addition to the overall and COVID-positive census counts, other variables were assessed based on either potential clinical importance or findings that these parameters varied throughout the study (and thus could be modeling predictors or confounders). The clinical variables of interest were general correlates of the weekly acuity: numbers of EMS arrivals, admissions, and highest-priority triage cases (*i.e.*, cases in the top two tiers of a five-tier triage system). The other variables assessed were demographic characteristics: sex, Qatari nationality, and age. These characteristics were primarily of interest as potential confounders; confounding was assessed by evaluating whether the inclusion of the parameter of interest resulted in a substantial change in the main variables’ effect magnitude.

### Initial bivariable model: Weekly census and weekly COVID-positive patient count

The initial model included two variables that were predefined as requiring inclusion: overall weekly census and the weekly number of COVID-positive patient diagnoses. The bivariable Poisson regression model, which incorporated the weekly number of EP shifts as an exposure (offset) term, yielded significant *p* values for patients’ weekly census (*p* = 0.024) and weekly numbers of new COVID-positive diagnoses (*p* < 0.001).

The bivariate model manifested a high value (1.21) for Pearson deviance, indicating residual dispersion. Further covariates were assessed in an attempt to reduce the Pearson deviance toward the target value of 1.0.^7^

### Assessment of ETI and NIV for inclusion

The next covariates of clinical interest were those dealing with airway and ventilation: ETI and NIV. The addition of ETI count (*i.e.*, total weekly ETIs) to the weekly counts of overall and COVID-positive patient *n* generated a trivariable Poisson regression model. In this model, the ETI variable was not significant (*p* = 0.616).

Unlike the case with ETI, the addition of NIV count (*i.e.*, total weekly NIV cases) to the bivariable model generated a finding of statistical significance (*p* = 0.025 for the NIV term). The decision was made to retain NIV in a trivariable model that included weekly all-patient count and weekly COVID-positive patient *n*.

The trivariable model had three statistically significant predictors, but the Pearson deviance (1.2) indicated remaining dispersion. Thus, additional parameters were assessed to control for patient acuity.

### Adjusting for patient acuity

These variables were available as markers of acuity. They described the arrival mode (EMS *vs.* other), triage into a high-acuity category (top two tiers of a five-tier system), and hospital admission. Neither EMS (*p* = 0.595) nor high-priority (*p* = 0.274) counts were statistically significant when added to the trivariable model.

The incorporation of weekly admissions (scaled as hundreds of admissions per week to facilitate IRR interpretation) resulted in a borderline *p* value of 0.054. However, the Pearson deviance of 1.0 (0.995 rounded) indicated that the admission *n* covariate was important. This importance was underlined because the four-variable model (weekly census counts, COVID-positive cases, NIV cases, and admits) had more favorable AIC and BIC than the trivariable model. The four-variable model had acceptable calibration by a postestimation goodness-of-fit test (*p* = 0.458).

### Defining the preliminary model

The modeling process at this point included four variables, representing weekly counts of all patients, COVID-positive cases, NIV cases, and admissions. Before this model, hereafter denoted the “four-variable model,” was accepted as the final model, three further steps were executed.

First, the ETI variable was explored to see whether it should supplement or replace the NIV variable. This exploration was indicated due to the importance of searching for any association between ETI and EP COVID conversion. In addition, the absence of such an association had important implications and was thus worth confirming.

Second, the demographic variables were explored to assess their need for inclusion in the model. The finding that these characteristics varied over the 21-week study warranted their investigation primarily as confounders.

Finally, Stata's *countfit* procedure was executed. This procedure allowed formal assessment regarding whether the Poisson regression model was associated with sufficient residual heterogeneity to warrant NB2 modeling. The *countfit* procedure also included an assessment regarding whether a zero-inflated modeling approach was required.

### Reintroduction of ETI back into the four-variable model

ETI was reintroduced into the four-variable model in two fashions. This approach was taken to maximize the chances of identifying any relationship between ETIs and EP COVID conversions.

First, ETI was introduced into the four-variable model as a replacement for the NIV variable. This resulted in a nonsignificant *p* value (0.696) for the ETI term.

Second, the ETI count was introduced into the four-variable model as an additional term. This also resulted in a nonsignificant *p* value (0.238).

Whether ETI replaced or was added to NIV counts, the ETI variable was not significant. These results support the conclusion that weekly counts of NIV, but not ETI, were associated with EP COVID conversions.

### Assessment of demographics

While there was little clinical rationale for the association between EP COVID conversions and weekly patients’ median age, sex, or nationality (assessed as Qatari *vs.* expatriate) counts. These factors were assessed to be conservative. The *p* values associated with introducing these variables into the four-variable model were not significant (age *p* = 0.078, male *p* = 0.650, Qatari *p* = 0.363).

In addition to the lack of statistical significance, the demographic variables did not exhibit confounding of the associations between other predictors in the four-variable model and EP COVID counts. Furthermore, Pearson deviance indicated worse dispersion when any of the three demographic covariates were added to the four-variable model.

### Final model evaluation: Evaluation of heterogeneity (NB2 α) and zero-inflated models

The Poisson regression model appeared to have acceptable performance with minimal residual dispersion. This was formally evaluated using the NB2 model and calculating a likelihood ratio test in which the overdispersion parameter (α) was equal to zero. The *p* value for this test was 0.500, indicating there was no evidence that the NB2 model was preferable to the Poisson regression model (which is essentially the same as an NB2 model with no residual overdispersion).

The lack of need for NB2 modeling was confirmed using Stata's *countfit* procedure, which based a recommendation for Poisson regression over NB2 on the nonsignificant *p* value for α. *Countfit* also reported a strong preference for Poisson regression over either zero-inflated Poisson or zero-inflated negative binomial modeling.

#### Ethics approval and consent to participate

This study protocol was approved by the Medical Research Center at Hamad Medical Corporation in Qatar. Informed consent was not applicable as the information was adequately anonymized.

#### Consent for publication

Not applicable.

#### Availability of data and materials

The datasets generated and/or analyzed during the current study are not publicly available due to anonymity but are available from the corresponding author on reasonable request.

#### Authors’ contributions list

**Shada A. Kodumayil**

Contributed data setup and analyses. Generated graphs and tables and contributed in the manuscript.

**Ashid Kodumayil**

Formed the statement of hypothesis and with the manuscript.

**Sarah A. Thomas**

Contributed data setup and analysis along with graphs and tables.

**Sameer A. Pathan**

Helped in formulating the research question. He also contributed in forming the statement of hypothesis and drafting of the initial manuscript.

**Zain A. Bhutta**

Contributed in data collection, analysis, and helped with the manuscript review.

**Isma Qureshi**

Contributed in data collection, analysis, and helped with the manuscript review.

**Aftab Azad**

Helped in formulating the research question and in forming the statement of hypothesis and drafting of the manuscript.

**Tim R. Harris**

Contributed in forming the concept and design of the manuscript.

**Stephen H. Thomas**

Contributed in forming the concept and design of the manuscript. Helped in data analysis and its interpretation. Provided the final approval for the manuscript to be sent in for publication.

### Funding

None

### Conflict of Interest

None for all Authors

## Figures and Tables

**Table 1:** Characteristics of ED patients seen in Qatar during the study period

Patient characteristics	Median (interquartile range) or % of 225,448
Age (median, interquartile range)	34 (27–43)
Male	75.9%
Qatari nationality	15.6%
Arrival by ambulance	27.2%
Highest two (of five) triage acuity categories	2.6%
Admitted to hospital (any unit)	11.3%

**Table 1a:** Demographics and acuity for n = 225,448 ED patients over 21 weeks

Week^*^	Daily census (mean ± SD^*^^*^)	% Arrival by ambulance	Age (median, IQR^*^^*^^*^)	% Male	% High-acuity^*^^*^^*^^*^	% Qatari nationality	% Admit

1	2305 ± 259	18.9%	32 (26–40)	69.8%	1.8%	16.9%	5.2%

2	1718 ± 423	25.8%	32 (26–40)	72.2%	1.7%	18.1%	6.2%

3	1259 ± 128	29.4%	33 (27–42)	71.3%	2.4%	20.4%	8.5%

4	1075 ± 106	30.4%	33 (27–43)	72.6%	2.4%	18.5%	9.0%

5	1203 ± 89	31.0%	34 (27–42)	76.4%	2.4%	16.8%	9.1%

6	1222 ± 116	33.0%	34 (27–43)	77.2%	3.8%	16.6%	10.2%

7	1395 ± 54	31.4%	34 (28–43)	79.2%	3.8%	14.9%	9.3%

8	1445 ± 76	33.5%	34 (28–43)	79.9%	3.5%	13.6%	10.1%

9	1592 ± 110	33.0%	34 (28–43)	81.0%	2.8%	12.3%	11.6%

10	1914 ± 130	27.5%	34 (28–42)	82.0%	2.8%	11.6%	11.6%

11	1885 ± 174	27.4%	34 (28–43)	81.0%	2.4%	11.1%	11.9%

12	1783 ± 217	27.4%	35 (28–43)	80.2%	2.7%	13.1%	13.9%

13	1727 ± 157	26.8%	35 (28–44)	78.6%	2.7%	13.4%	12.9%

14	1541 ± 174	24.8%	35 (28–43)	77.3%	2.3%	14.1%	12.2%

15	1511 ± 117	25.6%	35 (28–44)	75.5%	2.6%	15.8%	12.3%

16	1417 ± 124	26.2%	35 (28–44)	73.7%	3.1%	16.9%	12.3%

17	1349 ± 87	27.2%	35 (28–44)	71.8%	2.9%	17.8%	10.8%

18	1368 ± 102	27.5%	34 (27–44)	72.3%	2.6%	18.4%	11.7%

19	1487 ± 108	23.6%	34 (27–44)	73.1%	2.2%	18.3%	15.5%

20	1558 ± 158	24.2%	34 (28–43)	73.4%	2.3%	17.7%	17.2%

21	1454 ± 209	25.3%	34 (27–43)	73.3%	3.1%	16.9%	15.7%


^^*^^For all variables p < 0.001 for comparison across 21 study weeks

^^*^^*^^ SD - standard deviation

^^*^^*^^*^^ IQR - interquartile range

^^*^^*^^*^^*^^ Top two triage tiers in a five-tier system

**Table 2:** Factors predictive of weekly EP COVID conversions

Variable	Incidence rate ratio	95% confidence interval	*P*

Weekly census in thousands of patients	0.25	0.10–0.66	0.0003

Weekly hundreds of COVID+patients	1.20	1.09–1.33	< 0.0001

Weekly noninvasive ventilation cases	1.07	1.01–1.13	0.0187

Weekly hundreds of admitted patients	1.30	0.98–1.71	0.0544*


^^*^^Included in model based on favorable Pearson deviance and Akaike/Bayesian Information Criteria
